# Morphology, Crystallinity, and Molecular Weight of Poly(ε-caprolactone)/Graphene Oxide Hybrids

**DOI:** 10.3390/polym11071099

**Published:** 2019-06-28

**Authors:** Isabel Castilla-Cortázar, Ana Vidaurre, Bernabé Marí, Alberto J. Campillo-Fernández

**Affiliations:** 1 Centro de Biomateriales e Ingeniería Tisular, Universitat Politècnica de València, 46022 Valencia, Spain; 2 CIBER de Bioingeniería, Biomateriales y Nanomedicina (CIBER-BBN), 46022 Valencia, Spain; 3 Departamento de Física Aplicada-IDF, Universitat Politècnica de València, 46022 Valencia, Spain

**Keywords:** hybrids, polycaprolactone (PCL), graphene oxide (GO), molecular weight, morphology, FT-IR, biocompatibility

## Abstract

A study was carried out to determine the effects of graphene oxide (GO) filler on the properties of poly(ε-caprolactone) (PCL) films. A series of nanocomposites were prepared, incorporating different graphene oxide filler contents (0.1, 0.2, and 0.5 wt%) by the solution mixing method, and an in-depth study was made of the morphological changes, crystallization, infrared absorbance, molecular weight, thermal properties, and biocompatibility as a function of GO content to determine their suitability for use in biomedical applications. The infrared absorbance showed the existence of intermolecular hydrogen bonds between the PCL’s carbonyl groups and the GO’s hydrogen-donating groups, which is in line with the apparent reduction in molecular weight at higher GO contents, indicated by the results of the gel permeation chromatography (GPC), and the thermal property analysis. Polarized optical microscopy (POM) showed that GO acts as a nucleating point for PCL crystals, increasing crystallinity and crystallization temperature. The biological properties of the composites studied indicate that adding only 0.1 wt% of GO can improve cellular viability and that the composite shows promise for use in biomedical applications.

## 1. Introduction

GO is made by oxidating graphite to separate the platelets followed by ultrasound stirring [1,2,3,4]. It is a form of graphene with functional groups on the surface, such as carboxyl, hydroxyl, and epoxide groups, which can improve its dispersability in organic solvents [5]. As it can establish good interactions with organic polymers it also has enhanced processability [6,7]. The key aspect in obtaining hybrid materials is the proper dispersion of the reinforcement in the matrix, as well as an excellent interaction between the components [5,6,7,8,9,10,11,12,13,14,15]. Different methods have recently been proposed to process GO, including in situ polymerization, melt blending, and solution mixing, to disperse graphenes in polymer matrices [16]. The second of these methods includes adding a dispersing additive, followed by dissolving a matrix polymer in cosolvents and evaporating the solvent or using non-solvent coagulation to recover the composites. Although the particles are more dispersed than in melt processing, the particles may tend to reaggregate while the solvent evaporates. Melt blending’s main benefit is that it can synthesize intercalated nanocomposites formed of low or non-polar polymers [6]. The different processes are known to produce different nanofiller dispersion levels in the matrix and affect the polymer composite properties in different ways [17,18]. Sonication, or high-speed shearing, together with solution-phase mixing have been proposed to improve its dispersity and make GO more soluble in non-polar solvents [19].

Aliphatic polyesters have attracted a great deal of research interest due to their biodegradability and biocompatibility [20]. Poly(ɛ-caprolactone) (PCL) is fully biodegradable, biocompatible, and nontoxic to living organisms. Due to its rheological properties, almost any polymer processing method can be used to produce a variety of biomaterial applications, such as drug-delivery systems, sutures, wound dressings, fixation devices, and for dentistry and tissue engineering in the form of muscle, bone, cartilage, tendon, blood, nerve, skin, and cardiac tissues [21]. Several studies have prepared a wide range of PCL hybrids with GO or reduced graphene oxide (RGO) and identified their properties. For example, Kai et al. [20] used GO as a filler to enforce PCL and studied its effects on crystallization and mechanical properties. Biocompatible nanofibrous PCL and PCL/GO nanocomposite films have been prepared by electrospinning with varying GO concentrations to study their mechanical properties [22,23]. A series of PCL/GO and PCL/RGO composites with covalently attached GO and RGO have been synthetized to evaluate GO and RGO dispersion in the PCL matrix and its mechanical and electrical properties [24,25,26]. Other studies found PCL/GO composites to be useful for various biomedical applications [22,27,28]. Due to its peculiar atomic arrangements, GO has a strong adsorbing capacity which makes it suitable for use as a gene/drug delivery vehicle [29,30].

Biocompatibility studies have focused on a variety of methods to evaluate the resulting cytotoxic effects. Several authors have studied graphene oxide (GO) biocompatibility in mice in a series of biological assays and obtained significant pathological changes [31]. Jassim et al. studied the mechanisms by which 2D materials can interact with physiological barriers for potential diagnostic and therapeutic use [32]. Graphenes in a polymer matrix can be toxic to mammalian cells [8,9,10,11]. Reduced bacterial reproduction has been found on composite surfaces [33,34,35], which is important for biomaterial implantation procedures, in which infection is frequent [36]. Sydlik et al. found GO to be moderately compatible in vivo in subcutaneous and intraperitoneal tissue sites, an important issue for medical applications with the risk of inflammation due to foreign bodies [37].

In this study, a series of PCL/GO hybrids were prepared while subtly varying graphene oxide content in order to tune their physicochemical properties. Hybrids were produced by mixing and subsequent solvent casting method with dioxane as solvent. Scanning electron microscopy (SEM) and transmission electron microscopy (TEM) were used to study the morphology, molecular weight distribution was analyzed by GPC, the thermal behavior was studied by differential scanning calorimetry (DSC) and thermal gravimetric analyses (TGA). Interactions between PCL and GO and its effects on crystallinity were analyzed through Fourier-transform infrared spectroscopy (FT-IR). Crystal structure was studied by x-ray diffraction (XRD), and spherulite growth was assessed by POM. Finally, L-929 mouse fibroblasts were used to evaluate in vitro cell compatibility.

## 2. Experimental

### 2.1. Materials

Poly(caprolactone)(PCL) pellets Sigma-Aldrich (*M*_w_ = 70,000–90,000) were used without further purification. Dioxane solvent from Fisher and powdered graphene oxide from Graphenea were used as received.

### 2.2. Methods

#### 2.2.1. Sample Preparation

PCL/GO samples were prepared by solution mixing and subsequent solvent evaporation as follows: PCL was dissolved in dioxane and mixed with three solutions containing different amounts of GO dispersed in dioxane by ultrasound for 20 min (VWR Ultrasonic Bath USC600TH, VWR International, Leuven, Belgium). The resulting mixtures (0.1, 0.2, and 0.5% *w*/*w* GO/PCL) were mechanically stirred and returned to the ultrasound bath for 4 h. After sounding, the samples were placed under a vacuum pump in continuous extraction for 30 min at 40 °C to eliminate bubbles and left under vacuum for seven days to completely remove the solvent. The films were labeled according to GO content (wt%) as follows: PCL (neat PCL), PCL/GO-0.1 (0.1 wt% of GO), PCL/GO-0.2 (0.2 wt% GO), and PCL/GO-0.5 (0.5 wt% GO). The mean thickness of all the films was 2 mm. A balance (Mettler Toledo) with a sensitivity of 0.01 mg was used to weigh the samples.

#### 2.2.2. Morphology: SEM and TEM

SEM pictures of samples were taken by a JEOL JSM-5410 (Tokyo, Japan) scanning electron microscope to study the surface and cross section morphology of the dried samples after previous sputter-coating with gold at 10 kV of acceleration voltage and 15 mm working distance. For the TEM observations sample slices were obtained with a cryogenic Leica EM UC7 (Wetzlar, Germany) ultramicrotome equipped with the Leica EM FC7 low temperature sectioning system. Samples were sectioned with a DIATOME diamond knife at around −70 °C and mounted on a copper grid. Images were taken by a JEOL JEM-1010 transmission electron microscope at 100 kV.

#### 2.2.3. FT-IR Analysis

In this study the spectra were obtained from 64 scans taken at a rate of 1 scan/second at ambient temperature by means of an ALPHA FT-IR Spectrometer (Bruker, Ettlingen, Germany) in the wavenumber range of 400–4000 cm^−1^. Measurements were obtained by attenuated total reflectance spectroscopy (ATR, Bruker, Ettlingen, Germany) with a Platinum ATR single reflection diamond ATR accessory.

#### 2.2.4. POM

POM was applied to directly observe the crystallization of neat PCL and the hybrids. Thin films were obtained by dissolving each sample in TetraHydroFuran (THF) (2% *w*/*w*) and subsequent solvent evaporation on a cover microscope slide. The samples were subjected to thermal treatment with a Linkam THMS600 (Waterfield, UK) hot stage mounted on a polarized light microscope (NIKON Eclipse E600, Tokyo, Japan) and equipped with a TP93 control unit. The samples were heated to 80 °C followed by cooling to 40 °C at 40 °C·min^−1^ and maintained at this temperature to allow crystallization, which was monitored by a video camera connected to a computer.

#### 2.2.5. GPC

The sample weight average molar mass was measured by gel permeation chromatographer at 35 °C by a Waters Breeze GPC system with a 1525 Binary HPLC pump (Waters Corporation, Milford, MA, USA), a 2414 Refractive Index Detector and Styragel HR THF columns. THF eluent was applied at a flow rate of 1 mL·min^−1^. Shodex monodisperse polystyrene standards (Showa Denko K.K. Kawasaki, Japan) were used to obtain the calibration curve.

#### 2.2.6. Atomic Force Microscopy (AFM)

AFM was carried out on a Bruker MultiMode 8 SPM (Billerica, MA, USA) in tapping mode in air on a NanoScope V Controller and NanoScope Version 8.15 software and an antimony (n) doped silicon cantilever (Bruker) with a nominal force constant of 3 N·m^−1^ and resonance frequency of 75 kHz. The phase signal at the tip resonance frequency was zero, with a tapping frequency between 5% and 10% below the resonance frequency, drive amplitude of 270 mV and amplitude setpoint of 600 mV, with the amplitude setpoint/free amplitude ratio at 0.83.

#### 2.2.7. DSC

The sample thermal properties were determined in triplicate on an indium-calibrated Mettler Toledo differential scanning calorimeter (DSC, Perkin Elmer, Überlingen, Germany), with an initial heating scan of −10 to 100 °C, a cooling scan of 100 to −10 °C and another heating scan of −10 to 100 °C at a scan rate of 10 °C/min. Crystallinity was assumed to be proportional to the experimental heat of fusion of 139.5 J·g^−1^ for the 100% crystalline PCL, according to [38].

#### 2.2.8. XRD

The PCL/GO samples’ X-ray diffraction spectra were taken on a Rigaku Ultima IV X-ray diffractometer in the Bragg–Bentano configuration with the Kα radiation of a Cu anode from 2θ = 5°– 35° at a speed of 2°·min^−1^. The Debye–Scherrer equation [39].

(1) $$D=0.9\frac{\lambda }{\beta cos\theta }$$

where D is apparent particle size, β the full-width at half-maximum (FWHM) of the x-ray diffraction line (additional peak broadening) in radians, λ the wavelength, and θ is the angle between the incident ray and the scattering planes. In Equation (1) the 0.9 constant is partially dependent on the degree of crystal symmetry, as other research groups have found [40,41].

#### 2.2.9. TGA

Three replicates of approximately 4 mg samples were subjected to thermogravimetric analyses (TGA) on a TGA/DSC 2 STAR System (Mettler Toledo, Columbus, OH, USA) thermobalance, at 10 °C·min^-1^ from 30 to 1000 °C in a nitrogen atmosphere of 50 mL·min^−1^ to obtain inorganic content and thermal decomposition profiles.

#### 2.2.10. Cell Culture and Statistical Analysis

PCL, PCL/GO hybrids and latex (positive control) were sanitized by soaking the surfaces with pure ethanol (Sharlab, Barcelona, Spain) for 1 h, ethanol 70% for 1 h, ethanol 50% for 10 min, ethanol 30% for 10 min and washed 3 times with Dulbecco’s phosphate-buffered saline (DPBS) without Ca^2+^ and Mg^2+^ (Thermofisher, Madrid, Spain). 0.4 g of the sanitized materials were soaked in 2 mL of Dulbecco’s modified eagle medium (DMEM) (Gibco) 4.5 g·L^−1^, glucose supplemented with 10% *v*/*v* fetal bovine serum (FBS) (Life Technologies SA), and 1% *v*/*v* penicillin/streptomycin (100 U/mL/100 mg/mL) at 37 °C in 5% CO_2_ atmosphere for 24 h, hereinafter known as the extraction medium.

Mouse fibroblasts L929 (Sigma-Aldrich Quimica SL, Madrid, Spain), passage 16, were seeded on a 96 treated-multiwell plate. Then, 10^4^ cells/well were cultured in the medium described above for 3 h to allow the cells to attach to the bottom surface. The medium was replaced by the extraction medium and the cells were incubated at 37 °C in a 5% CO_2_ atmosphere for 48 h. Treated-polystyrene wells were used as negative control.

Cell vitality was evaluated by replacing the extraction medium by a solution of DMEM without phenol-red with 10% of methylthiazolyldiphenyl-tetrazolium bromide (MTT) and incubated at 37 °C in 5% CO_2_ for 2.5 h. The cells were then washed twice with DPBS without Ca^2+^ and Mg^2+^ (Sigma-Aldrich) and a solubilization solution of isopropanol (Sigma-Aldrich), 0.1% *v*/*v* Nonidet P 40 Substitute (Sigma-Aldrich), and 4 mM HCl (Sharlab) for 30 min in an orbital shaker to break the cell membranes and extract the formazan crystals. Spectrophotometric quantification at a wavelength of 570 nm was performed on a Victor 31,420 Multilabel counter spectrophotometer (Perkin Elmer, Turku, Finland). The mean value and statistical deviation of the obtained absorbance were obtained from six replicates after subtracting the mean value of the blank (without cells). The nonparametric Kruskal–Wallis test was applied to the materials, plus a post hoc Mann–Whitney U-test on paired groups if significant differences were found.

## 3. Results and Discussion

### 3.1. Visual Examination, SEM, and TEM

**Figure 1:**
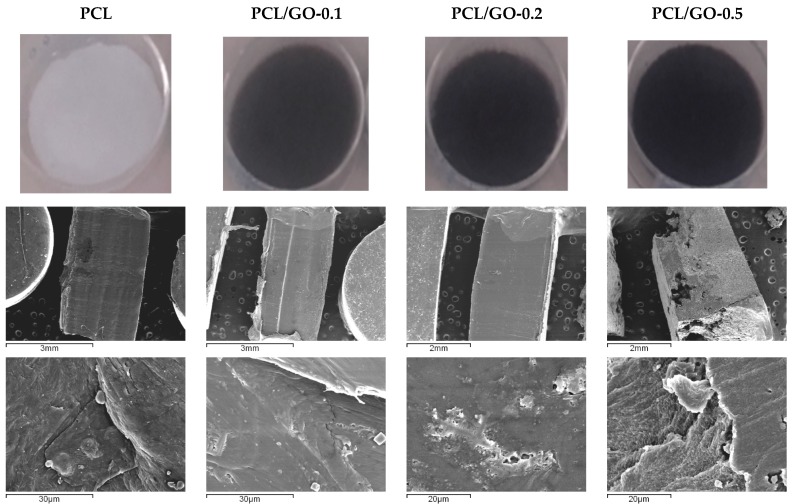
Sample photographs (above) and transversal SEM images according to GO content.

Pristine PCL was seen to be white by visual examination, while hybrid samples showed a uniform dark color, which become deeper as GO concentration increased (Figure 1). The microstructure of the surfaces and cross sectional area of the prepared films and their surfaces appeared practically smooth and even when monitored by SEM, with the exception of the PCL/GO-0.5 samples, which had a different structure. These samples had a flat, smooth upper surface, but an inconsistent porous distribution in the matrix, although no pores were visible on their surface. Similar morphological differences have been found elsewhere on opposite faces of PCL films prepared by solvent evaporation [42]. The surface in contact with the glass was found to be rough and had pores forming irregular voids, while the surface open to the air was less porous and smoother.

**Figure 2:**
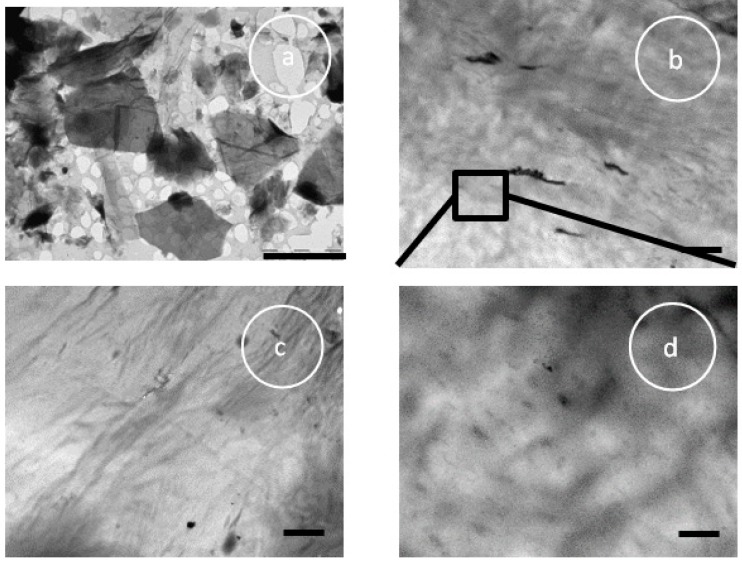
TEM images of GO (**a**); PCL/GO-0.2 (**b**); PCL/GO-0.5 (**c**) and PCL/GO-0.2 at higher magnification (**d**); Scale bar for (**a**) represents 5 microns, for (**b**,**c**) 2 microns and for (**d**) 500 nm.

The size dispersion of the GO flakes can be seen in Figure 2a after passing through the ultrasonic bath dispersion process. The largest flake is around 4 microns together with fragments in the order of nanometers. Figure 2b,c show TEM images of the hybrids PCL/GO-0.2 and PCL/GO-0.5, respectively, where the sheets are seen to be heterogeneously dispersed in the matrix, together with areas with GO aggregates. Figure 2d shows a higher magnification of Figure 2b with GO sheets of different sizes dispersed in the matrix.

### 3.2. POM

**Figure 3:**
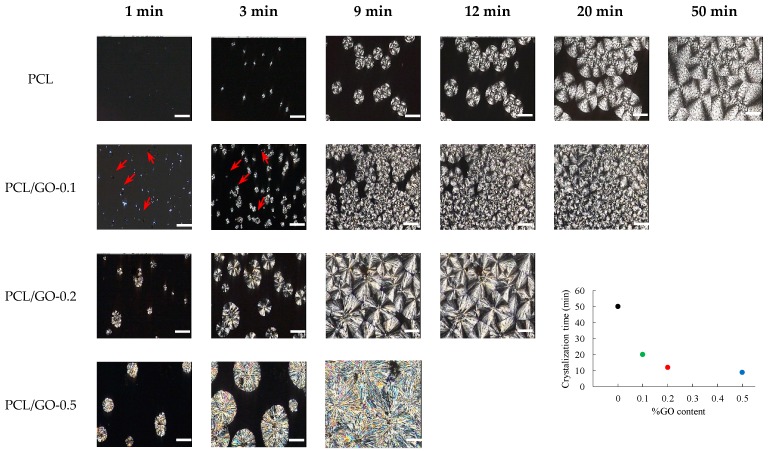
Spherulite growth according to GO content (wt%). Graph shows the time at which crystallization is completed vs. GO content (wt%) (white bar represents 100 microns).

**Figure 4:**
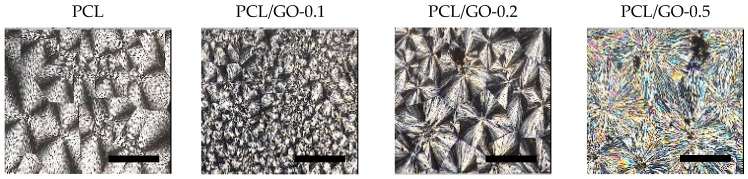
Spherulite final structure according to GO content (wt%) (black bar represents 200 µm).

POM images are shown in Figure 3 and Figure 4. The isothermal growth of spherulites at 40 °C can be observed as a function of time and GO content. Firstly, it should be noted that PCL shows a structure with an average grain size of ~0.02 mm^2^; when 0.1 wt% GO is added the average grain size decreases sharply, while the trend reverses with higher contents, with larger average size as GO increases, as can be seen in Figure 3. It is worth noting that GO acts as a nucleation point for the spherulites: The red arrows for PCL/GO-0.1 represent GO fragments, which act as nuclei for spherulite growth. At higher contents GO tends to stack with fewer and larger crystals with large defects on the grain boundaries. This could explain the extreme fragility of the PCL/GO-0.5 samples, as they break easily, which is in agreement with the mechanical properties observed in previous studies [23]. Crystallization time (crystals covering the whole frame) was found to diminish with GO content, as shown in Figure 3, from ~50 min for neat PCL to ~9 min for PCL/GO-0.5, which seems to obey a negative exponential law. This again can be attributed to the GO acting as a nucleation point. In order to better observe the final structure, Figure 4 shows the spherulites after the crystallization process. The evolution of crystal size with the previously mentioned GO content can be clearly seen.

### 3.3. FT-IR

**Figure 5:**
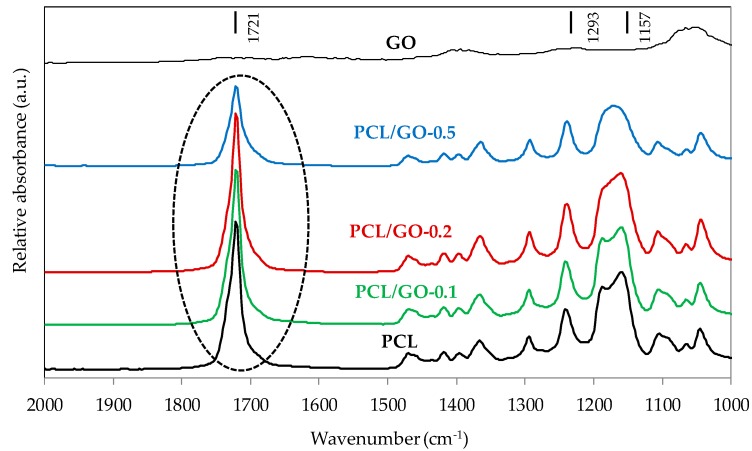
FT-IR spectra of GO, neat PCL and PCL/GO composites.

FT-IR spectra of GO, neat PCL and PCL/GO composites are shown in Figure 5. A prominent characteristic absorption at 1,721 cm^−1^ for neat PCL and hybrids corresponding to the carbonyl stretching mode can be seen.

**Figure 6:**
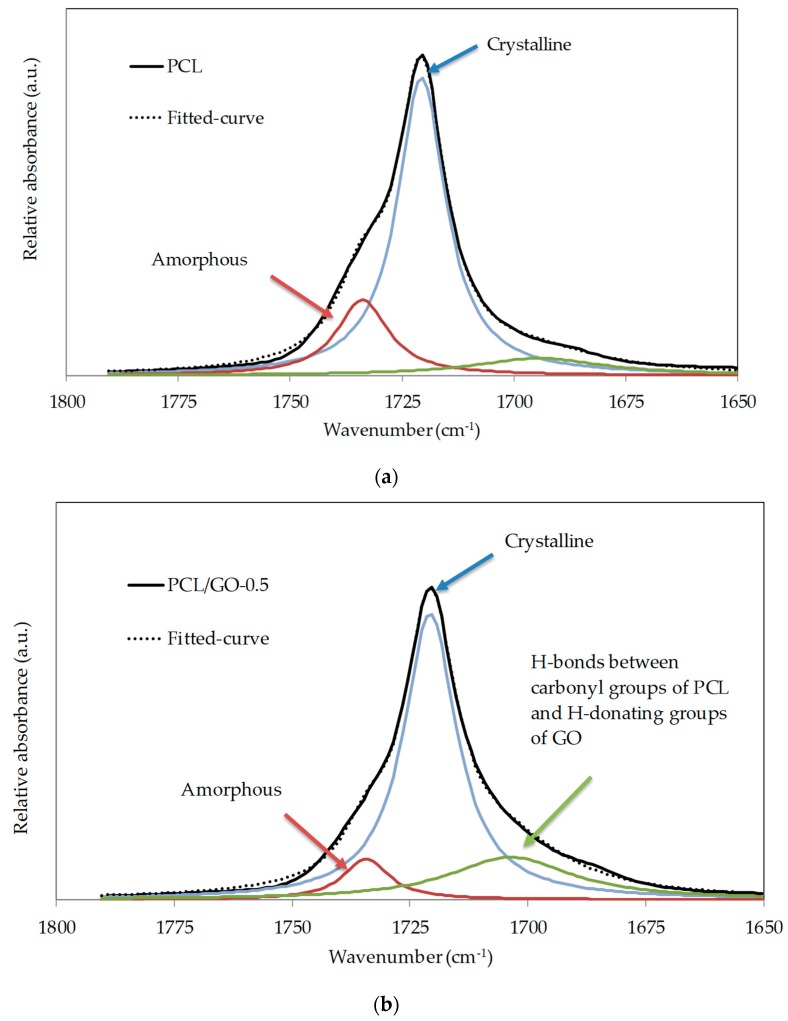
Experimental and fitted-curve FT-IR spectra of the C=O stretching region of (**a**) PCL (**b**) PCL/GO-0.5.

Due to the semicrystalline nature of PCL and its composites, spectra were resolved on the basis of three Laurentzian shaped absorption bands with maximum absorbance at 1734, 1721, and 1700 cm^−1^, which are attributed to carboxyl absorption bands of amorphous phase, crystalline phase, and hydrogen bonds [22,43,44]. The peak at 1700 cm^−1^ was due to hydrogen-bonded carbonyl vibration thought to be caused by the intermolecular hydrogen bonds between the PCL carbonyl groups and GO hydrogen-donating groups [22]. Figure 6a,b show the FT-IR spectrum and the fitted curves of the neat PCL and the hybrid PCL/GO-0.5, respectively. For the sake of comparison, numerical results of the normalized intensity of the bands at 1734, 1721 and 1700 cm^−1^ are shown in Table 1. The peak around 1700 cm^−1^ increases with GO content, showing the growth of intermolecular hydrogen bonds as GO content increases. It should also be noted that the amorphous peak decreases as the GO content increases.

Likewise, the absorption of the C–C and C–O groups was analyzed in the crystalline and amorphous phases, corresponding to the 1293 and 1157 cm^−1^ wavelengths, respectively (see Figure 5). It is important to mention that two peaks can be distinguished for neat PCL at 1180 and 1157 cm^−1^, while three bands are overlapping [45]. Also, as GO content increases these two peaks become a single broad peak, indicating the decrease of the amorphous phase Table 2 shows the absorbance intensity at 1293 and 1157 cm^−1^, where, again, the ratio between the crystalline and amorphous phases increases with GO content.

### 3.4. GPC

**Figure 7:**
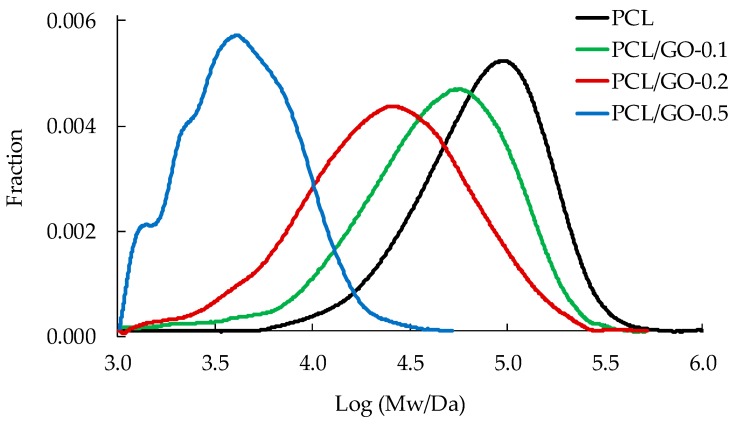
Molecular weight distribution according to GO content (wt%).

Figure 7 shows the molecular weight distribution obtained by GPC using the Mark–Houwink–Sakurada parameters provided by Huang et al. [46], (k = 2.9·10^−4^ (dL·g^−1^) α = 0.7). Table 3 gives the weight-average molecular weight, *M*_w_, and polydispersity index (PDI) for the samples with different GO contents. As can be seen in Figure 7, *M*_w_ was unexpectedly found to drop significantly when the GO content was added, although the procedure followed to prepare the samples by solution mixing and solvent evaporation should not affect the PCL molecular weight. In other studies [24,44,45] with polymerization in the presence of GO it has been found that GO reduced the initiator efficiency and the reaction rate, with a slight increase in the average molecular weight of the polymer formed, but this was not the case here. The GPC measurements were repeated with samples of different molecular weights prepared with PCL, and other samples prepared following the same procedure, but with THF instead of dioxane as solvent. The results showed the same tendency to reduce the measured *M*_w_ as GO content increased.

Many of the processes by which fillers affect polymers are still not fully understood, as has been pointed out in the literature [47,48]. The simulation approach in these studies focuses on the ways the filler particles change both the distribution of the end-to-end distances of the polymer chains and the radius of gyration. The extent to which the filler particles can change polymer properties depends significantly on the chain size *R*_pol_ vs. particle size *R*_part_. In simulations, as long as the energetic filler-polymer interaction is not unrealistically high, the main results are quantitatively governed by the polymer-filler size ratio Ψ=RpolRpart finding three different regimes:Ψ<1,Ψ≈1andΨ>1 [48]. Some of the simulations find a smaller average radius of gyration *R*_g_ when Ψ≈1 and Ψ>1. To our knowledge, no studies have yet been published specifically on the conformations of GO–PCL nanocomposites.

**Figure 8:**
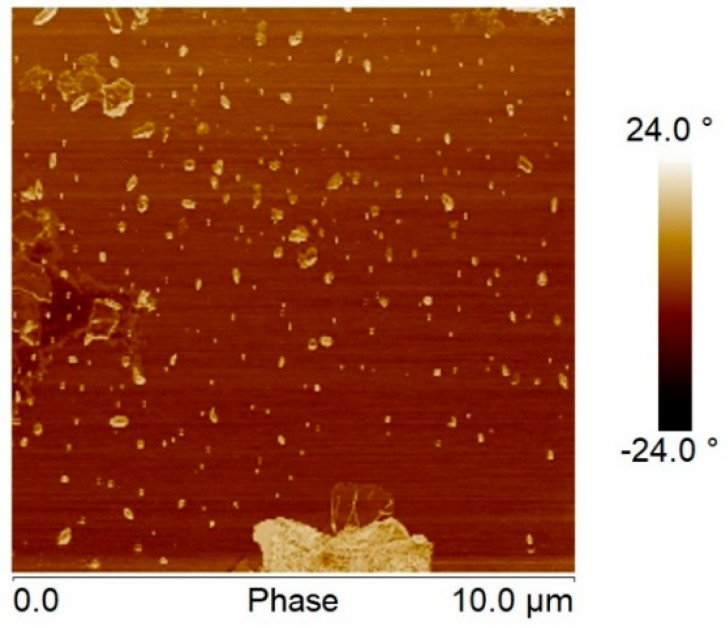
AFM phase image of GO flakes deposited onto mica.

**Figure 9:**
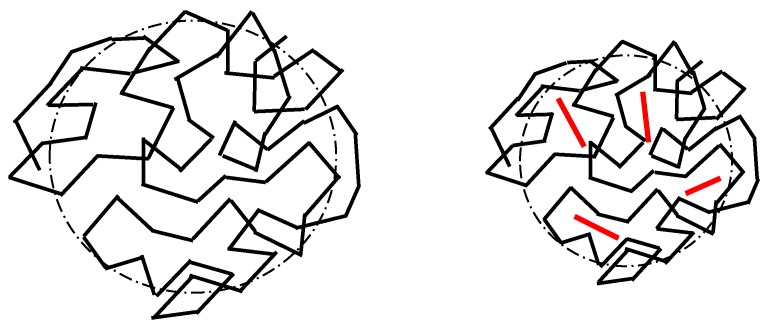
Schematic representation of neat PCL (left) and PCL/GO (right) in a good solvent.

It should be remembered that when analyzing our hybrid samples, the GO flakes varied widely in size, from a few nanometers to microns, as can be seen in Figure 2a and Figure 8. It is also estimated that the end-to-end distance of the dissolved macromolecule should be around 10 nm, and the radius of gyration around 4 nm (taking into account a Flory’s characteristic ratio, C_∞_, equal to 5 and an average bond length of 0.149 nm [46]), so that the size of the GO nanoplates inserted into the macromolecules must be of that order or smaller (Figure 9).

Based on the above-mentioned information and the way in which the GPC works to obtain molecular weight distribution, we tried to find an explanation for the reduction in molecular weight we obtained. It is known that the macromolecular chains of neat PCL are highly compact, especially in the crystalline zones. When the sample is immersed in a good solvent, e.g. THF, the solvent molecules penetrate into the core of the chains and the crystals are dissolved. Under these conditions, a very low-concentration solution passes through the GPC columns and the elution time is as shown in Figure 7 (black profile). On the other hand, when a PCL/GO hybrid is produced, nano-GO flakes are entrapped by macromolecular chains as the solvent evaporates, and different (secondary) interactions between nano-GO and macromolecular chains could take place due to the hydroxyl, carboxyl and epoxy groups present in the GO. When the hybrid is dissolved in THF before the GPC analysis, solvent penetration is counterbalanced by the expansion of the macromolecules, which finds greater resistance due to non-covalent interactions between nano-GO and PCL macromolecules. This could be responsible for the reduced chain dimensions (see Figure 9) [48,49], and therefore longer elution time, and an apparent reduction in the molecular weight as measured by GPC compared to neat PCL. Duan et al. [50] also found that the molecular weight of the PCL matrix decreased with increasing GO content, but they attributed it to the degradation of PCL chains. We find our explanation more plausible than that of Duan et al. since we tested the molecular weight immediately after sample preparation and found the same effect. In addition, Duan et al. do not give any data on the molecular weight evolution with time, but only provide the GPC results 50 days after sample preparation.

The effect is more pronounced as the percentage of GO increases (see Table 3). The samples containing the highest GO content (see Figure 7) present a complex distribution of molecular weight that seems to consist of three overlapping peaks, indicating the presence of three main distributions, possibly due to molecules with different amounts of GO trapped inside. Polydispersity was found to increase with GO content, except for the hybrid PCL/GO-0.5, which was lower than pure PCL (see Table 3).

### 3.5. DSC

**Figure 10:**
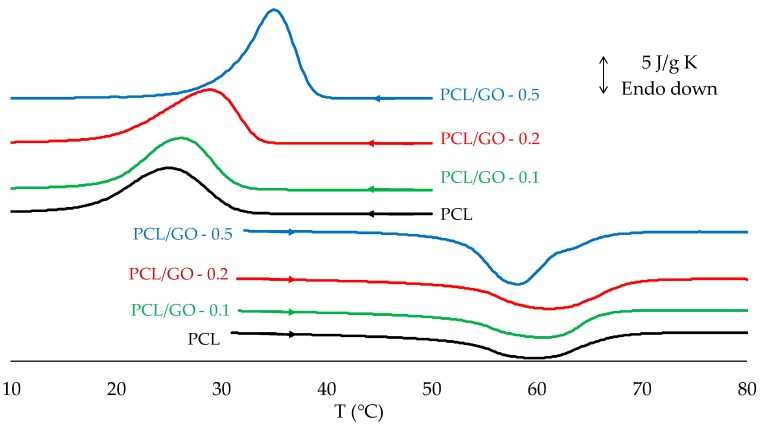
DSC scans for PCL/GO hybrids on cooling and second heating scan (after removing thermal history).

The changes in thermal properties according to GO content were monitored by DSC. Figure 10 shows the DSC curves after removing the thermal history (second heating) and cooling.

**Figure 11:**
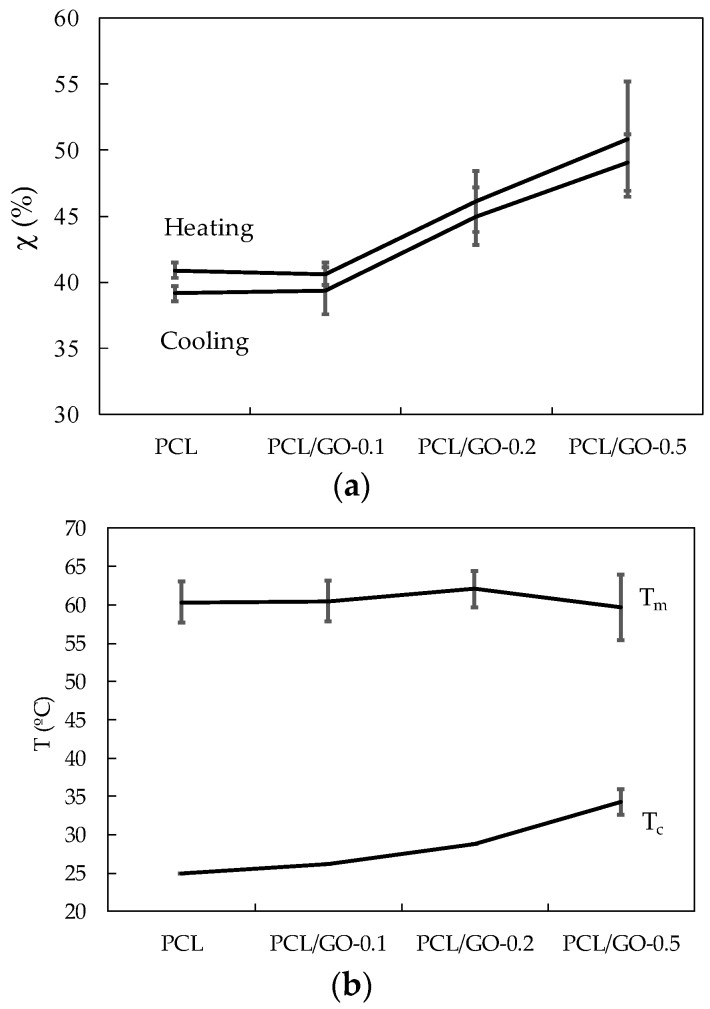
(**a**) Effect of GO on hybrid crystallinity. (**b**) Effect of GO on crystallization and melting temperatures.

Figure 11 shows crystallinity, χ, (Figure 11a), crystallization peak temperature on cooling, *T*_c_, and melting peak temperature of the second heating scan, *T*_m_ (Figure 11b). It can be seen that crystallinity rose steadily when GO was added, from 39.1% ± 1.8% for pure PCL to 49.0% ± 3.1% for PCL/GO-0.5, a remarkable relative increase of 25%, as has been reported in previous studies [24,28,51]. The increase in crystallinity with GO can be attributed to the nucleating action of GO sheets in the PCL matrix, as has been observed through POM (Figure 3). Some studies have shown that graphene increases the number of crystallization nucleation sites and thus changes the size and number of the spherulite crystalline regions in polycaprolactone [25]. However, other studies [20,52] have found that crystallinity is reduced when the volume fraction of the fillers is increased, which they attribute to the interfacial interactions between GO nanoplatelets and PCL molecular chains. This reduces the chain flexibility and retards the crystallization process. This is not so in our case, since the GO nanosheets could serve as heterogeneous nuclei for PCL crystallization, suggesting that the higher GO content could have raised the crystallinity, as can be seen in (Figure 11a) and was observed by FT-IR. It should also be noted that the samples containing 0.5 wt%, GO have a much broader melting peak (see Figure 10) and there seem to be various peaks combined into one, which suggests that GO nanoplatelets have enlarged the size spectrum of the crystals.

DSC data show that adding GO to the PCL structure did not significantly affect the melting point of the hybrids with respect to neat PCL (Figure 11b), as previously reported in [51,53]. The fact that the melting temperature of PCL/GO hybrids is found to be almost unchanged indicates that adding graphene (GO) did not significantly affect the PCL melting behavior. The higher Tc (Figure 11b) when GO was added could mean that the dispersed GO nanosheets acted as efficient nucleating seeds and promoted PCL crystallization, as was seen by POM (Figure 3 and Figure 4).

### 3.6. XRD

**Figure 12:**
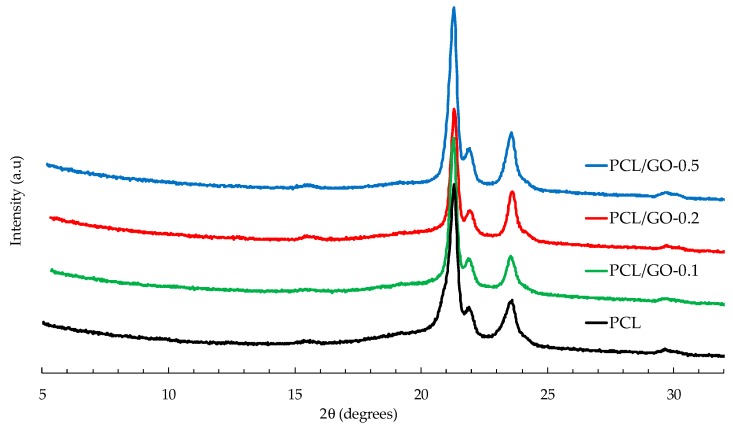
X-ray diffraction of neat PCL and PCL/GO hybrids.

Figure 12 represents the X-ray diffraction profiles for hybrid samples of PCL/GO. The diffraction pattern shows characteristic PCL peaks at 2θ = 21.3° and 23.6° for pristine PCL, which correspond to (110) and (200) crystallographic planes, respectively [54]. No GO diffraction peaks can be seen for the analyzed PCL/GO samples with different GO loadings, indicating that adding GO has no significant effect on the crystal structure of the PCL matrix [51,55,56,57] and does not significantly affect the XRD patterns, except for a slight increase in the crystallinity of the PCL/GO hybrids, similar to the effect observed by DSC and FT-IR. The lack of GO diffraction peaks could be attributed to the proper dispersion of GO nanosheets within the PCL matrix, as reported in previous studies [58,59].

**Figure 13:**
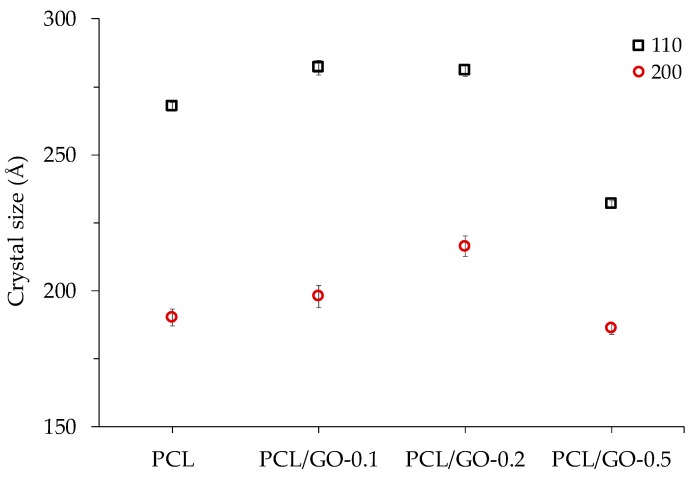
Crystal size of the 110 and 200 planes for different samples.

The apparent crystal size was obtained from FWHM by applying Equation (1) to the two main peaks. The results shown in Figure 13 indicate a tendency to larger crystal sizes for 0.1 and 0.2 wt% GO content and smaller sizes for the composite containing 0.5 wt% GO. This last result, also found in a previous study [28], can be attributed to the effect of the physical barrier composed of nanoparticles that limits crystallite size.

### 3.7. TGA

**Figure 14:**
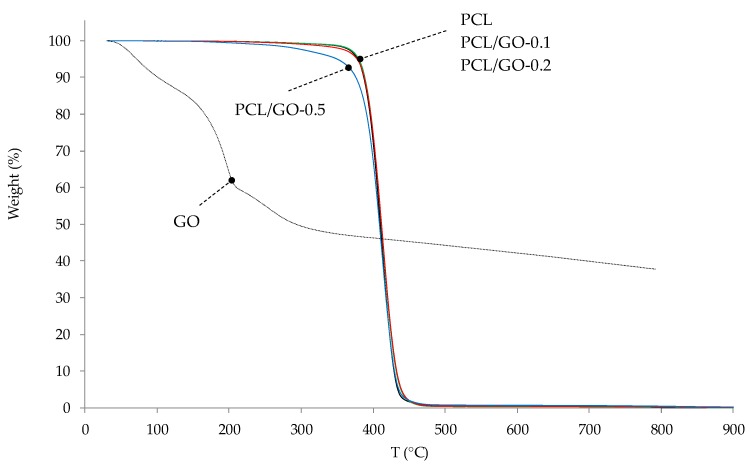
TG plots of PCL and PCL/GO nanocomposites.

**Figure 15:**
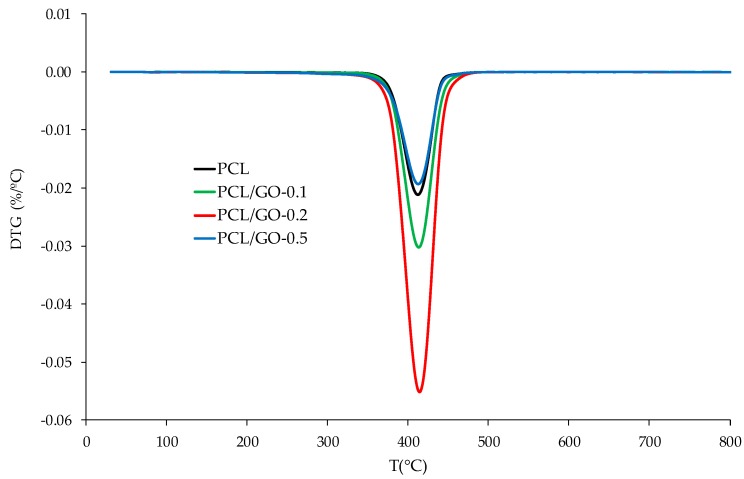
Derivative thermogravimetric (DTG) curves of PCL and its nanocomposites.

TGA was used to analyze composite thermal stability, as can be seen in Figure 14 and Figure 15. Initial decomposition temperature (IDT) (5% weight loss temperature), *T*_max_ (max degradation rate temperature), residue at 800 °C and the theoretical residue obtained by the typical behavior of an ideal mixture are given in Table 4. This criterion is used to check whether there are any interactions between both components. Equation (2) was used to calculate the theoretical residue of each sample. The GO thermal degradation data were taken from [60].

(2) $$\frac{\Delta m}{m_{0}}=w_{PCL}\cdot \frac{\Delta m_{PCL}}{m_{0,PCL}}+\left(1-w_{PCL}\right)\cdot \frac{\Delta m_{GO}}{m_{0,GO}}$$

As observed in Table 4, the hybrids’ IDT remains fairly constant with GO contents of up to 0.2 wt%, whereas PCL/GO-0.5 has an IDT around 30 °C lower than neat PCL, which could be related to the loss of labile oxygen GO groups.

The derivative thermogravimetric (DTG) curves (Figure 15) of neat PCL and its nanocomposites were found to undergo single-stage degradation at approximately 413 °C. The maximum degradation rate temperature (*T*_max_) of PCL and its hybrids did not change, indicating that GO does not affect polymer mass diffusion.

Equation (2) predicts that residues at 800 °C should be 0.14%, 0.18%, and 0.29% for PCL/GO-0.1, PCL/GO-0.2, and PCL/GO-0.5, respectively, although the real residues are higher than those predicted, as can be seen in Table 4. This agrees with the previous results and indicates an interaction between PCL and GO, probably due to secondary bonds between the polymer chains and GO flakes during solvent evaporation.

### 3.8. Cellular Viability

**Figure 16:**
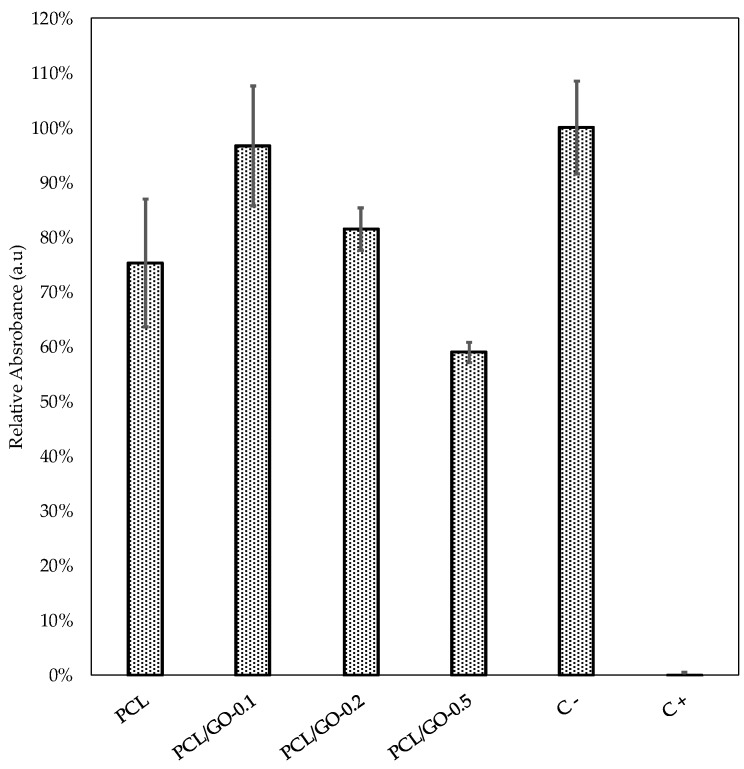
Relative absorbance of the MTT analysis as a function of GO composition. Values are presented as mean ± SD from six replicates at 48 h. Kruskal–Wallis test shows significant differences between materials (k = 22.4473; p = 0.00016). Mann–Whitney U-test showed significant differences between PCL and PCL/GO-0.1 ((U = 1; p = 0.0083), PCL and C- (U = 1; p = 0.0083), PCL /GO-0.1 and PCL/GO-0.2 (U = 2; p = 0.01314), PCL/GO-0.1 and PCL/GO-0.5 (U = 0; p = 0.00512), PCL /GO-0.2 and PCL/GO-0.5 (U = 0; p = 0.00512), PCL /GO-0.2 and C- (U = 2; p = 0.01314), and PCL /GO-0.5 and C- (U = 0; p = 0.00512), whereas no significant differences were found between PCL and PCL/GO-0.2 ((U = 12; p = 0.37886), PCL and PCL/GO-0.5 (U = 6; p = 0.06576), PCL/GO-0.1 and C- (U = 14; p = 0.57548).

L-929 mouse fibroblasts were used to study PCL and PCL/GO hybrid biocompatibility and the biological response was analyzed by MTT. Figure 16 shows the results after 2 days of culture. The higher absorbance value is in proportion to the number of viable cells. In this study, the cells grown on different materials were compared with polystyrene (negative control), assigning 100% viability to the latter. PCL, PCL/GO-0.1, and PCL/GO-0.2 were above 70% in cell vitality and could be considered non-toxic for the L929 cell line, according to ISO 10993, while the mean value of absorbance for cells in contact with PCL/GO-0.5 was 58.9% ± 1.9%. Some studies have found that the GO toxic effect on cells depends on the dose used, and that lower GO concentrations affect cell behavior positively [23,61], phenomena which were also found in the present study. The biological response results showed that adding GO in small doses not only did not restrain cell proliferation but in fact facilitated it. When the GO concentration reached 0.5 wt%, it had adverse effects on cellular behavior, as has also been found by other authors [23].

## 4. Conclusions

PCL films incorporating GO at different filler contents (0.1, 0.2 and 0.5 wt%) were produced by the solution mixing method to advance the understanding of PCL/GO hybrid materials and consider their suitability for use in biomedical applications. 

We identified the interactions between the reinforcement, GO, and the matrix, PCL, by means of different techniques. For instance, FT-IR revealed that hydrogen-bonds could have arisen between GO’s H-donating groups and PCL’s carbonyl groups. POM clearly revealed that the crystallization time is reduced at higher GO concentrations, confirming GO’s nucleation effect. The interactions between GO and PCL were also confirmed by the DSC and TGA results. The PCL/GO hybrids were found by DSC to have slightly higher crystallinity and crystallization temperature than neat PCL, which confirms GO’s good nucleating effect on PCL crystallization. TGA showed that the real residues were higher than those predicted for an ideal mixture, which again indicates an interaction between the components. The XRD results showed that adding GO does not significantly affect the XRD patterns, except for a slightly higher hybrid crystallinity, similar to the effect observed by DSC.

It should be noted that the GPC data showed a marked reduction of molecular weight with GO content, which can be attributed to the apparently smaller macromolecule size, probably due to the GO nanoflakes being trapped inside the PCL chains and secondary interactions taking place between the GO nanoflakes and polymer chains.

Finally, the cell viability test on L-929 mouse fibroblasts showed that there are statistical differences between PCL/GO-0.1 and the rest of the hybrids, indicating that adding only 0.1 wt% of GO can improve cellular viability and that the composite shows promise for use in biomedical applications. The next step in this line of research will be to evaluate hydrolytic degradation as a potential candidate for tissue engineering applications.

## Figures and Tables

**Table 1:** Normalized intensity of the carboxyl absorption band decomposition at 1734, 1721 and 1700 cm^−1^.

Sample	Crystalline	Amorphous	H-Bonding
PCL	76.3%	19.3%	4.4%
PCL/GO-0.1	77.1%	18.0%	4.8%
PCL/GO-0.2	77.4%	14.0%	8.5%
PCL/GO-0.5	77.4%	11.1%	11.5%

**Table 2:** Absorbance intensity of the C–C and C–O groups in the crystalline and amorphous phase at 1293 and 1160 cm^−1^.

Sample	1293 cm^−1^ Crystalline	1157 cm^−1^ Amorphous	Crystalline/Amorphous Ratio
PCL	0.19667	0.53359	37%
PCL/GO-0.1	0.22069	0.56913	39%
PCL/GO-0.2	0.25354	0.57757	44%
PCL/GO-0.5	0.22497	0.49367	46%

**Table 3:** *M*_w_, and PDI, according to GO content (wt%).

Sample	*M*_w_	PDI
PCL	97095	1.63
PCL/GO-0.1	61472	1.77
PCL/GO-0.2	37878	2.66
PCL/GO-0.5	4436	1.59

**Table 4:** Thermal properties of PCL and PCL/GO nanocomposites. Initial decomposition temperature, IDT, temperature of maximum degradation rate, *T*_max_, residue measured at 800 °C, and theoretical residue at the same temperature.

Sample	IDT (°C)	*T*_max_ (°C)	Residue at 800 °C (%)	Theoretical Residue at 800 °C (%)
PCL	378.3	412.2	0.10	
PCL/GO-0.1	379.9	413.3	0.17	0.14
PCL/GO-0.2	377.5	414.4	0.27	0.18
PCL/GO-0.5	350.2	412.6	0.45	0.29
